# Development of 11-DGA-3-*O*-Gal-Modified Cantharidin Liposomes for Treatment of Hepatocellular Carcinoma

**DOI:** 10.3390/molecules24173080

**Published:** 2019-08-24

**Authors:** Lili Zhou, Manshu Zou, Kun Zhu, Shuangcheng Ning, Xinhua Xia

**Affiliations:** School of Pharmacy, Hunan University of Chinese Medicine, Changsha 410208, China

**Keywords:** targeted liposomes, cantharidin, 3-galactosidase-30-stearyl deoxyglycyrrhetinic acid, asialoglycoprotein receptor, glycyrrhetinic acid, hepatocellular carcinoma

## Abstract

Background: Liver cancer is a common malignant tumor worldwide, and its morbidity and mortality increase each year. The disease has a short course and high mortality, making it a serious threat to human health. Purpose: The objective of this study was to create novel liver-targeting nanoliposomes to encapsulate cantharidin (CTD) as a potential treatment for hepatic carcinoma. Methods: 3-Galactosidase-30-stearyl deoxyglycyrrhetinic acid (11-DGA-3-O-Gal)-modified liposomes (11-DGA-3-O-Gal-CTD-lip) for the liver-targeted delivery of CTD were prepared via the film-dispersion method and characterized. In vitro analyses of the effects on cellular cytotoxicity, cell migration, cell cycle, and cell apoptosis were carried out and an in vivo pharmacokinetics study and tissue distribution analysis were performed. Results: Compared with unmodified liposomes (CTD-lip), 11-DGA-3-O-Gal-CTD-lip showed higher cytotoxicity and increased the inhibition of HepG2 cell migration, but they did not increase the apoptotic rate of cells. The inhibition mechanism of 11-DGA-3-O-Gal-CTD-lip on hepatocellular carcinoma was partly through cell cycle arrest at the S phase. Analysis of pharmacokinetic parameters indicated that 11-DGA-3-O-Gal-CTD-lip were eliminated more rapidly than CTD-lip. Regarding tissue distribution, the targeting efficiency of 11-DGA-3-O-Gal-CTD-lip to the liver was (41.15 ± 3.28)%, relative targeting efficiency was (1.53 ± 0.31)%, relative uptake rate was( 1.69 ± 0.37)%, and peak concentration ratio was (2.68 ± 0.12)%. Conclusion: 11-DGA-3-O-Gal-CTD-lip represent a promising nanocarrier for the liver-targeted delivery of antitumor drugs to treat hepatocellular carcinoma.

## 1. Introduction

As a major public health concern worldwide, cancer has received widespread attention from all parts of society, but the burden of cancer will increase in coming decades, especially in low- and middle-income countries (LMIC) [1,2]. Although the fight against cancer has been continuous, the mortality rate for various cancers has only decreased by 2% [3]. Primary liver cancer, comprised majorly of hepatocellular carcinoma (HCC) and intrahepatic cholangiocarcinoma, had become the 5th most common malignant tumor, the 2th cause of cancer death worldwide in 2018 [4]. HCC is one of the most common malignant tumors worldwide, and its morbidity and mortality increase each year [5,6]. HCC has a short course of disease and high mortality, presenting a serious threat to human health. Chemotherapy and surgery remain the main treatments to date [7]. Most anti-cancer drugs used for cancer treatment are highly toxic and have poor specificity [8]. Moreover, tumor cells are prone to developing resistance to chemotherapeutic drugs, which also leads to poor therapeutic effects [9]. Therefore, there is an urgent need to develop a new treatment plan for liver cancer.

Cantharides, which is the dry body of *Mylabris phalerata Pallas* or *Mylabris cichorii* Linnaeus, is the first insect-based drug with an anti-tumor effect discovered in China. Cantharidin (CTD) is the main active ingredient of cantharides. Modern pharmacological studies have shown that cantharidin has inhibitory effects on various cancers such as liver cancer (especially primary liver cancer) [10], oral cancer [11], pancreatic cancer [12], gastric cancer [13,14], breast cancer [15], osteosarcoma [16] and lung cancer [17]. Moreover, its effects include recruiting white blood cells, stimulating bone marrow formation [18], and not inhibiting the immune system [19]. However, cantharidin is highly toxic, and clinical studies have shown that oral and intravenous cantharidin have serious effects on the urinary system and digestive system, which can lead to severe gastric ulcer disease, intraepithelial ulcers in urinary organ tubules, and intraperitoneal cavity capillary congestion, followed by severe liver congestion and edema [20]. Because of its strong toxicity, cantharidin can readily cause poisoning or even death [21], and its serious side effects greatly limit the application of cantharidin for treating tumors.

In recent years, there has been rapid development in the use of liposomes as drug delivery systems, especially as a carrier of anti-tumor drugs [22]. After entering the body, they are mainly phagocytized by the reticuloendothelial system, so that the drugs mainly end up in the liver, spleen, lungs, and bone marrow. Although the drug accumulates in certain tissues and organs [23,24], liposomes fail to deliver precise targeting at the organ or cell level. To further improve drug efficacy, reduce side effects, and improve stability, many scholars have attempted to functionally modify the surface of liposomes [25]. Commonly used specific targeting materials include polysaccharides and their derivatives [26], targeting molecules combined with ligands (folate combined with folate receptor, transferrin combined with transferrin receptor) [27,28], and peptides [29], which can result in active targeting and improve the treatment effect.

The asialoglycoprotein receptor (ASGPR) is an expression-rich endocytic receptor on the liver cell membrane that recognizes sugar chains with galactose residues or N-acetylgalactosamine groups at the ends [30,31]. By targeting this receptor, a drug can be specifically introduced into hepatocytes to treat diseases such as hepatitis B and hyperlipidemia [32] or directly delivered into liver cancer cells to treat primary liver cancer [33,34]. Galactosylated liposomes have been shown to significantly improve the liver targeting of pharmaceutical preparations [35].

The glycyrrhetinic acid (GA) binding site has strong affinity for GA and is found in high abundance on the liver cell membrane, making GA an important biofilm factor on hepatocyte membranes [36,37]. There are binding sites for GA and glycyrrhizic acid on the liver cell membrane, but some studies have confirmed that the expression of the GA receptor is much higher than that of the glycyrrhizic acid receptor [38]. Compared with normal hepatocytes, liver cancer cells more readily recognize and bind GA, and GA can be used as a modification material to specifically target liver cancer sites, thereby reducing the accumulation of drugs in normal liver tissues [39]. However, studies have shown that the long-term use of GA can produce similar side effects as aldosterone, which can lead to adrenaline-like hyperkinesia with symptoms such as hypertension, hypokalemia, and sodium retention. The side effects of GA have been shown to decrease after the reduction of the 11th carbonyl group of GA to a methylene group [40].

In this study, a single ligand double-target liposome targeting the ASGPR and GA receptor was developed for the effective delivery of cantharidin. The synthesized novel target molecule 11-DGA-3-O-Gal can guide the liposome to actively target hepatocellular carcinoma cells. On the one hand, it can improve the shortcomings of cantharidin, such as insolubility in water and toxicity, and on the other hand, it can improve the selectivity of drugs, reduce the damage to other tissues and organs, and thereby enhance efficacy and reduce toxicity. In addition to characterizing the physicochemical properties of the modified liposomes, we also evaluated their effects on cytotoxicity, cell migration, the cell cycle, and apoptosis and examined their pharmacokinetics and biological distribution in vivo.

## 2. Results and Discussion

### 2.1. Synthesis and Structure Confirmation of 11-DGA-3-O-Gal

**Figure 1:**
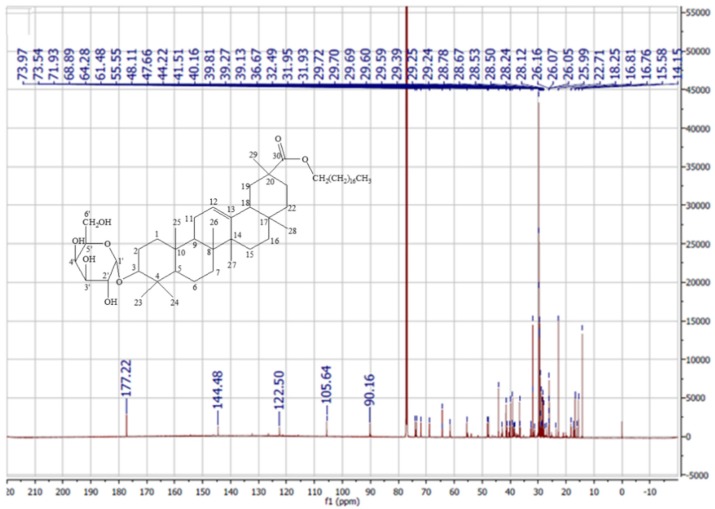
Structure and ^13^C-nuclear magnetic resonance (NMR) spectra of 11-DGA-3-O-Gal.

11-DGA-3-O-Gal was synthesized successfully by esterification reaction in organic media. Both synthesis steps were monitored by thin-layer chromatography (TLC) to ensure complete the reaction. The molecular weight detected was 893.69 [M + Na]^+^ in positive-ion mode, which suggested that the product obtained was identical with 11-DGA-3-O-Gal (molecular weight: 870.69). The ^13^C-nuclear magnetic resonance (NMR) spectrum of 3-acetylation-galactosidase-30-stearyl deoxyglycyrrhetinic acid showed that the chemical shift values at the C3 atom was shifted to a lower magnetic field (δ79.03→δ90.92), which indicated esterification proceeded between DGA and acetobromo-α-d-galactose. The ^13^C-NMR spectrum of 11-DGA-3-O-Gal (Figure 1) showed that the peaks of δ177.55, 170.53, 170.38, 169.48 disappeared, which indicated that deacetylation was successful. The purified DGA, 3-acetylation-galactosidase-30-stearyl deoxyglycyrrhetinic acid and 11-DGA-3-O-Gal were analyzed by mass spectrometry (MS) and NMR spectroscopy. The data is shown below:

DGA: electrospray ionization (ESI)-MS *m*/*z*: 731.63 [M + Na]^+^. ^13^C-NMR(600 MHz, CDCl_3_): δ177.28(C-30), δ144.53(C-13), δ122.48(C-12), δ79.03(C-3), δ64.29(C-1″), δ55.19(C-5), δ48.22(C-9), δ47.64(C-18), δ44.23(C-20), δ42.85(C-14), δ41.55(C-19), δ39.78(C-8), δ38.79(C-4), δ38.60(C-1), δ36.95(C-10), δ32.66(C-22), δ31.31(C-7), δ29.69(C-17), δ29.39(C-21), δ29.29(C-28), δ28.79(C-23), δ28.66(C-16), δ28.23(C-2), δ28.11(C-15), δ27.25(C-27), δ26.97(C-11), δ18.37(C-29), δ16.79(C-6), δ15.60(C-25), δ15.52(C-26), δ14.15(C-24). ^1^H-NMR (600 MHz, CDCl_3_): δ3.23(1H, m, H3), δ5.25(1H, t, *J* = 3.01 Hz, H12), δ1.12, 1.11, 0.99, 0.94, 0.87, 0.79, 0.77(3H, s, -CH_3_)

3-acetylation-galactosidase-30-stearyl deoxyglycyrrhetinic acid: ESI-MS *m*/*z*: 1061.73 [M + Na]^+^. ^13^C-NMR (600 MHz, CDCl_3_): δ177.36(C-30), δ177.55(-COCH_3_), δ170.53(-COCH_3_), δ170.38(-COCH_3_), δ169.48(-COCH_3_), δ144.66(C-13), δ122.61(C-12), δ103.62(C-1′), δ90.92(C-3), δ71.14(C-4′), δ70.54(C-3′), δ69.38(C-2′), δ67.23(C-5′), δ61.49(C-6′), δ64.42(C-1′’), δ55.65(C-5), δ48.32(C-9), δ47.76(C-18), δ44.36(C-20), δ42.98(C-14), δ41.66(C-19), δ39.93(C-8), δ39.19(C-4), δ38.69(C-1), δ38.49(C-10), δ32.78(C-22), δ32.07(C-7), δ29.82(C-17), δ29.72(C-21), δ29.51(C-28), δ29.39(C-23), δ28.92(C-16), δ28.79(C-2), δ28.34(C-15), δ27.88(C-27), δ27.09(C-11), δ18.38(C-29), δ16.90(C-6), δ16.53(C-25), δ15.57(C-26), δ14.27(C-24). ^1^H-NMR(600MHz, CDCl_3_): δ3.11(1H, dd, *J* = 11.48,4.80 Hz, H3), δ5.25(1H, t, *J* = 4.81 Hz, H12), δ1.12, 1.11, 0.95, 0.93, 0.87, 0.77, 0.76 (3H, s, -CH3), δ5.37(1H, d, *J* = 3.48 Hz, H1′), δ5.02(1H, dd, *J* = 3.48, 10.50 Hz, H2′), δ5.26(1H, dd, *J* = 10.50, 11.16 Hz, H3′), δ4.21(1H, dd, *J* = 11.16, 6.78 Hz, H4′), δ4.11(1H, m, H5′), δ3.89(1H, dd, *J* = 10.26, 5.76 Hz, H6′a), δ4.52(1H, dd, *J* = 10.26, 2.15 Hz, H6′b),δ2.14, 2.04, 2.04, 1.98(3H, s, -OC-CH_3_).

11-DGA-3-O-Gal: ESI-MS *m*/*z*: 893.69 [M + Na]^+^. ^13^C-NMR(600 MHz, CDCl3): δ177.22(C-30), δ144.48(C-13), δ122.50(C-12), δ105.64(C-1′), δ90.16(C-3′), δ73.97(C-4′), δ73.54(C-5′), δ71.93(C-2′), δ61.48(C-6′), δ68.89(C-3), δ64.33(C-1″), δ55.55(C-5), δ48.11(C-9), δ47.66(C-18), δ44.22(C-20), δ42.82(C-14) δ41.51(C-19), δ39.81(C-8), δ39.27(C-4), δ39.13(C-1), δ36.67(C-10), δ31.95(C-22), δ31.93(C-7), δ29.72(C-17), δ29.70(C-21), δ29.69(C-28), δ29.25(C-23), δ29.24(C-16), δ28.78(C-2), δ28.67(C-15), δ28.24(C-27), δ28.12(C-11), δ18.25(C-29), δ16.81(C-6), δ16.76(C-25), δ15.58(C-26), δ14.15(C-24). ^1^H-NMR(600 MHz, CDCl_3_):δ3.20(1H, dd, *J* = 11.36, 4.77 Hz H3), δ5.29(1H, t, *J* = 4.74 Hz, H12), δ1.15, 1.13, 1.05, 0.98, 0.90, 0.86, 0.79(3H, s, -CH_3_), δ5.29(1H, d, *J* = 3.55 Hz, H1′), δ4.18(1H, dd, *J* = 3.36, 10.57 Hz, H2′), δ3.71(1H, dd, *J* = 10.52, 11.19 Hz, H3′), δ3.65(1H, dd, *J* = 11.20, 6.83 Hz, H4′), δ3.54(1H, m, H5′), δ4.05(1H, dd, *J* = 10.26, 5.82 Hz, H6′a), δ4.14(1H, *J* = 10.26, 2.18 Hz, H6′b)

### 2.2. Characterization of Liposomes

**Figure 2:**
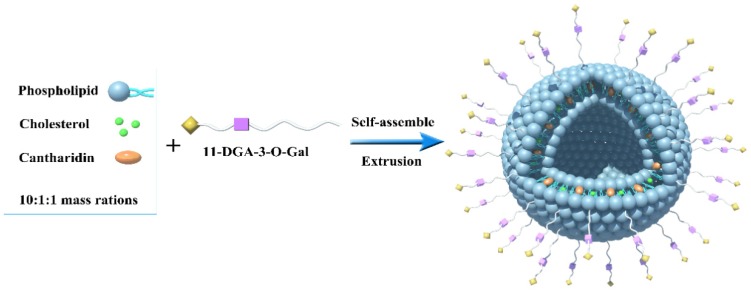
Schematic diagram of 11-DGA-3-O-Gal-CTD-lip. CTD was encapsulated into the hydrophobic shell of the liposomes. 11-DGA-3-O-Gal was inserted into the phospholipid bilayer of the liposomes.

**Figure 3:**
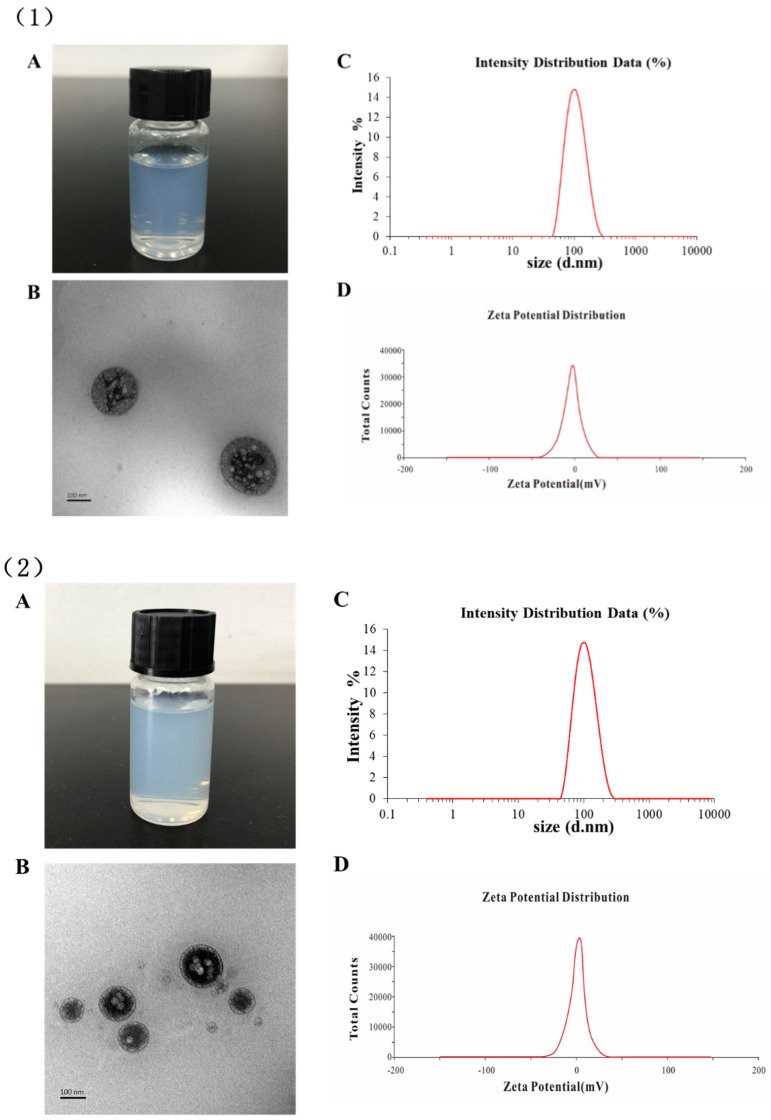
Appearance, transmission electron photomicrographs, size distributions, and zeta potentials of CTD-lip (**1**) and 11-DGA-3-O-Gal-CTD-lip (**2**). (**A**) Appearance; (**B**) transmission electron photomicrographs; (**C**) size distribution; (**D**) zeta potential distribution.

Both types of liposomes (11-DGA-3-O-Gal-CTD-lip and CTD-lip) were prepared using the film dispersion method (Figure 2). Visual examinations revealed that the lipid films were uniform and delicate, and the liposome solution obtained appeared clear and transparent with light blue opalescence. The size distribution, polydispersity index (PDI), and zeta potential (ZP) of the different liposomes are shown in Table 1. All liposomes had a narrow size distribution and relatively high entrapment efficiency. Images of liposome morphology were obtained by transmission electron microscopy. A single factor test was designed based on the appearance, particle size, and encapsulation efficiency of the liposomes. The ratio of targeting molecule-lipid (1:15, 1:10, 1:7.5, 1:6) added to the cantharidin liposome solution was investigated. As the amount of targeting molecule increased, the turbidity and particle size of liposomes increased. In the range of a single factor investigation, when the ratio was 1:10, the liposomes were clear and transparent with pale blue opalescence and a stable particle size, and the encapsulation efficiency was greater than 90%. No significant changes were observed in any characteristic following the addition of 11-DGA-3-O-Gal to the liposomes. The appearance, morphology, size distribution, and zeta potential distribution are presented in Figure 3.

### 2.3. In Vitro Release of Cantharidin (CTD)

**Figure 4:**
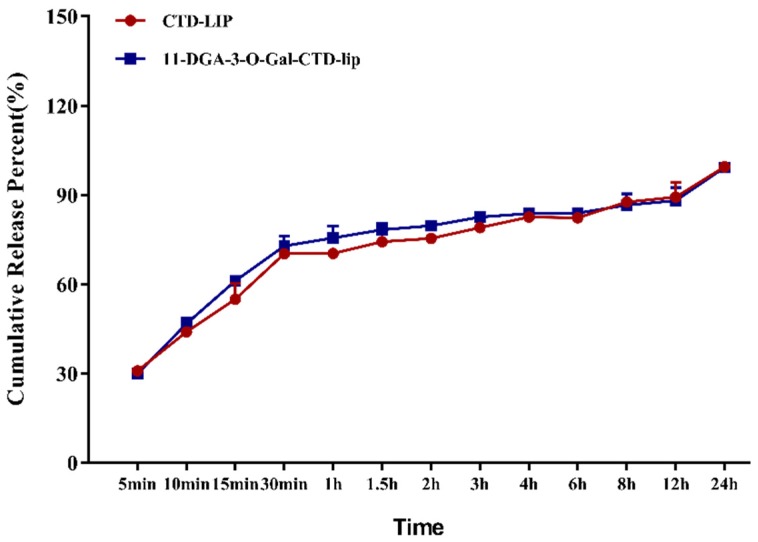
In vitro release profiles of CTD from liposomes in phosphate-buffered saline (PBS, pH = 7.4, 0.25% Tween-80). Data are expressed as the mean ± standard deviation (SD, n = 3).

The in vitro release of CTD from CTD-lip and 11-DGA-3-O-Gal-CTD-lip was studied, and this experiment was carried out in phosphate-buffered saline (PBS, pH = 7.4) with 0.25% Tween-80. As shown in Figure 4, after encapsulation of CTD into liposomes, the two nano-formulations showed a similar drug release profile, with ~70% released in an initial burst followed by a slow phase. Both reached ~90% of cumulative drug release after 24 h. The release curve of CTD in the liposomes conformed to the Weibull equation, as shown in Table 2. These results indicated that the addition of targeting molecules had no significant effect on the release of liposomes, which will be beneficial for the pharmacodynamics processes in the body.

### 2.4. In Vitro Cytotoxicity Assay

**Figure 5:**
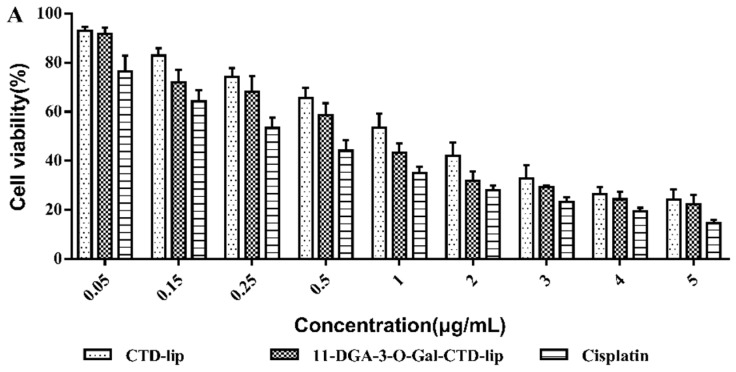

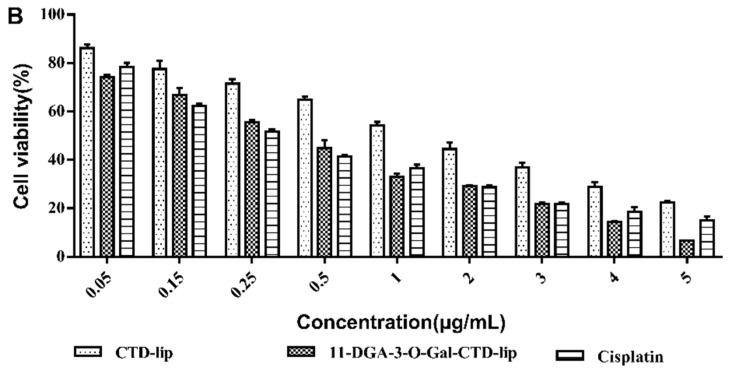
In vitro cytotoxicity of the various liposomal formulations on (**A**) HepG2 cells and. (**B**) L-02 cells. Data are presented as the mean ± SD (n = 6).

The cytotoxic effects of various liposomal formulations in HepG2 and L-02 cells are shown in Figure 5. The results indicated that both CTD-lip and modified-CTD-lip inhibited the proliferation of HepG2 and L-02 cells, and the inhibitory effect on L-02 cells was stronger than that on HepG2 cells. Under the same experimental conditions, the HepG2 cytotoxicity results indicated that the IC_50_ value of 11-DGA-3-O-Gal-CTD-lip was 0.772 ± 0.021 μg/mL, and compared with that of CTD-lip, the inhibitory effect on HepG2 cell proliferation was 1.64 times higher.

### 2.5. Cell Migration Assays

**Figure 6:**
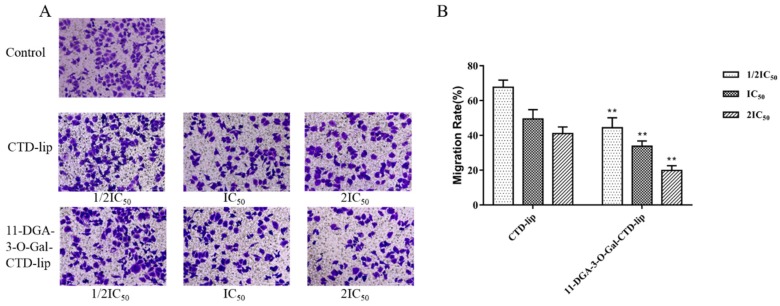
Inhibition of cancer cell migration was evaluated using cell migration assays with HepG2 cells. (**A**) Cell migration assays at 24 h after treatment with various formulations observed at 10 × 10 magnification. (**B**) Migration ratios were measured using Image-J. ** *p* < 0.01, 11-DGA-3-O-Gal-CTD-lip vs CTD-lip.

Transwell chemotaxis assays were used to determine whether the liposomal formulations affected the migration abilities of HepG2 cells. Tumor cells showed differences in migration abilities after treatment with the different CTD formulations (Figure 6A). CTD-lip slightly inhibited the migration of cells compared with the control group, with migration rates of (67.1 ± 4.1)%, (49.4 ± 5.4)%, and (41.0 ± 3.8)% at 1/2IC_50_, IC_50_, and 2IC_50_ concentrations, respectively. CTD-lip also played a minimal role in inhibiting tumor cell migration. However, 11-DGA-3-O-Gal-CTD-lip showed a stronger migration inhibition ability, with rates of (44.3 ± 5.8)%, ( 33.8 ± 3.0)%, and (19.9 ± 2.7)% at 1/2IC_50_, IC_50_, and 2IC_50_ concentrations, respectively. This indicated that modification of liposomes by 11-DGA-3-O-Gal resulted in a stronger migration inhibitory ability than that of CTD-lip. Compared with the inhibition by CTD-lip, the inhibition of HepG2 cell migration ability by 11-DGA-3-O-Gal-CTD-lip was 1.52 times, 1.46 times, and 2.06 times higher at the different concentrations (Figure 6B). The results of the cell migration test were consistent with the cytotoxicity test results, in which 11-DGA-3-O-Gal-CTD-lip showed the highest toxicity. 11-DGA-3-O-Gal increased the active targeting of liposomes and increased the cellular uptake of drugs. Moreover, 11-DGA-3-O-Gal-CTD-lip inhibited the migration of cancer cells significantly.

### 2.6. Inhibition Mechanism of 11-DGA-3-O-Gal-CTD-Lip in HepG2 Cells

**Figure 7:**
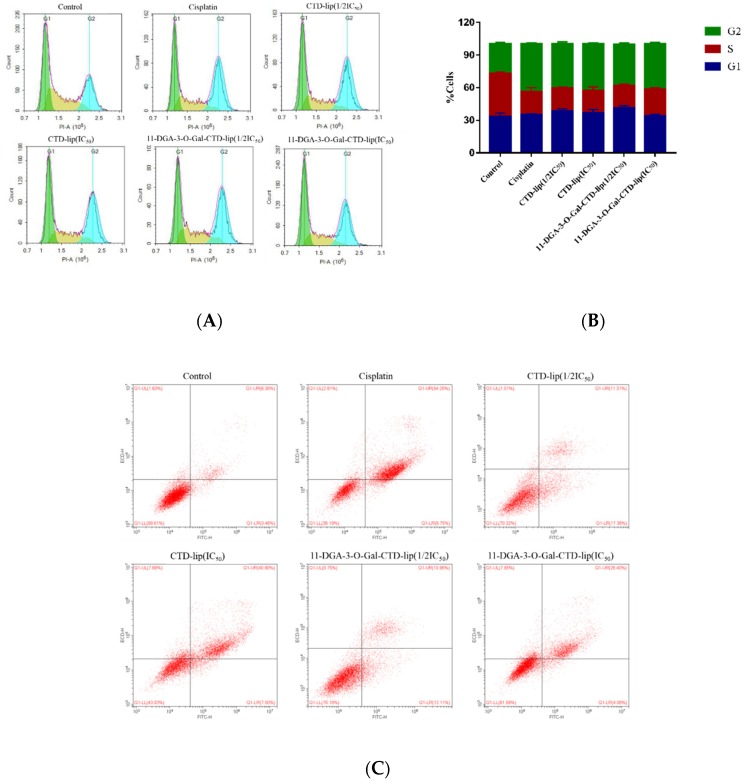

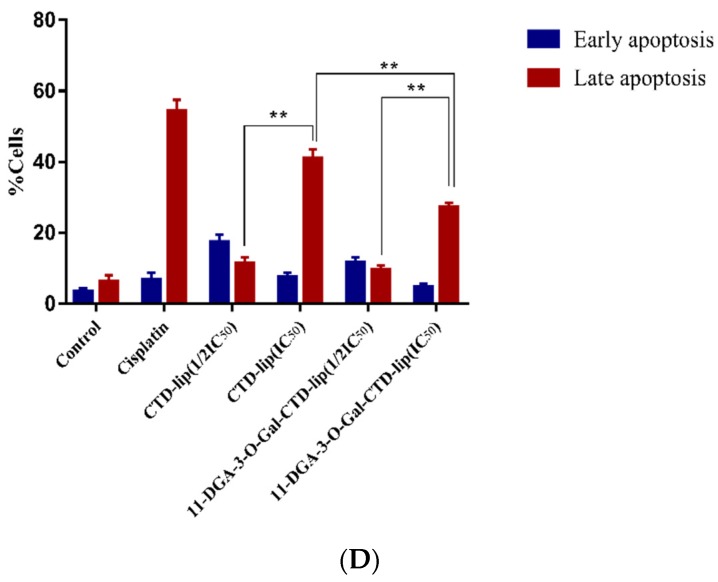
The mechanism of 11-DGA-3-O-Gal-CTD-lip inhibiting HepG2 cell growth. (**A**) Cell-cycle distribution of HepG2 cells after treatment with 11-DGA-3-O-Gal-CTD-lip and non-targeted CTD-lip at a CTD concentration of IC_50_ and 1/2IC_50_. (**B**) Stacked plot showing the percentage of HepG2 cells in each phase of the cell cycle. Data are presented as the mean ± SD (n = 3). (**C**) Apoptosis evaluation in HepG2 cells using flow cytometry after treatment with different CTD formulations at a CTD concentration of IC_50_ and 1/2IC_50_. (**D**) Quantitative data of HepG2 cells for early apoptotic cells and late apoptotic cells attained by flow cytometry. Data are presented as the mean ± SD (n = 3). ** *p* < 0.01.

A rapid proliferation rate is a prominent feature of tumor cells. Therefore, inhibition of the occurrence and development of tumor cells and promotion of tumor cell apoptosis are among the relevant factors to be considered for anti-tumor drugs. We investigated the effect of 11-DGA-3-O-Gal-CTD-lip on the cell cycle and apoptosis using flow cytometry. The results showed that the percentage of cells in G2 phase of the cell cycle significantly increased from (27.10 ± 1.99)% to (41.72 ± 1.79)% after treatment with 11-DGA-3-O-Gal-CTD-lip compared to 42.62 ± 1.09% after treatment with CTD-lip at the IC_50_ concentration (Figure 7A,B). The results were similar at the 1/2IC_50_ concentration, suggesting that 11-DGA-3-O-Gal-CTD-lip induced the accumulation of HepG2 cells in S phase of the cell cycle. Moreover, the cell apoptosis results indicated that the cells treated with 11-DGA-3-O-Gal-CTD-lip had a lower apoptotic rate (31.36 ± 1.14)% than the cells treated with CTD-lip (50.68 ± 3.06)% at the IC_50_ concentration (Figure 7C,D). Compared with the blank control group, the proportion of early apoptosis and late apoptosis of cells treated with CTD liposomes increased significantly, and the difference in the apoptosis rate at each concentration group was statistically significant (*p* < 0.01). Comparison of the 11-DGA-3-O-Gal-CTD-lip group with the CTD-lip group revealed that the apoptosis rate of the liposomes did not significantly change after the modification with the targeting molecule, indicating that the effect of CTD liposomes on the apoptosis of HepG2 cells may be independent of the addition of the targeting molecule.

### 2.7. In Vivo Pharmacokinetics Study

**Figure 8:**
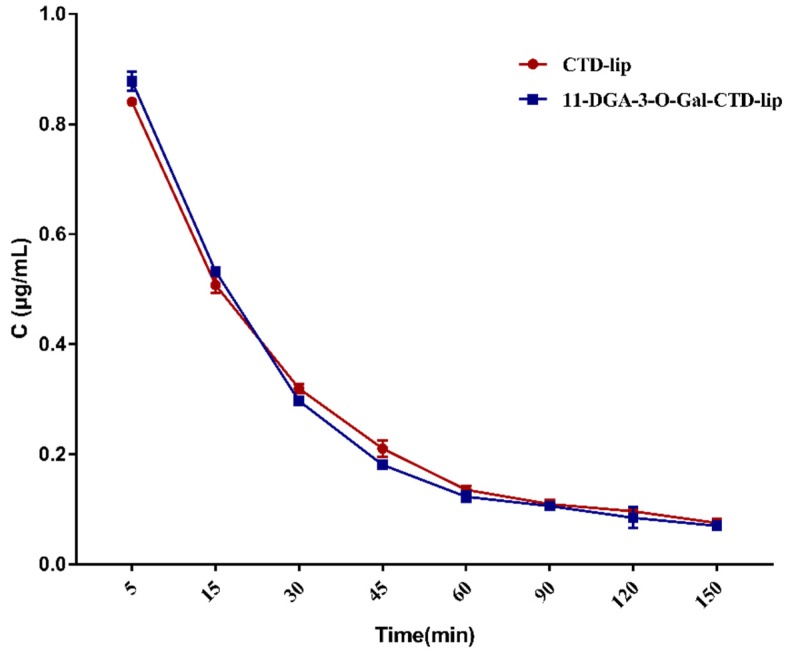
Mean plasma concentration of CTD in rats after intravenous administration (mean ± SD, n = 6).

The pharmacokinetic properties of 11-DGA-3-O-Gal-CTD-lip and CTD-lip were studied by detecting the CTD content in rat plasma. The mean plasma concentration-time curves of CTD after intravenous administration of 11-DGA-3-O-Gal-CTD-lip and CTD-lip are shown in Figure 8.

The drug-time curve trends of the two liposomes were similar, and the drug was eliminated faster in the first 60 min, which may be related to the rapid elimination of free drugs from the liposome preparation and the liposome burst effect. After 60 min, the elimination of the drug in the blood slowed down. A non-compartment model was suitable to evaluate the plasma drug concentration time curves obtained in rats based on the analysis of models and parameters. The main pharmacokinetic parameters are summarized in Table 3. Compared with that of CTD-lip, the elimination half-life (T_1/2β_) of 11-DGA-3-O-Gal-CTD-lip decreased, the value of which was 2.42 ± 1.03 h. The results indicated that 11-DGA-3-O-Gal-CTD-lip could rapidly distribute to tissues from the blood. The elimination rate of 11-DGA-3-O-Gal-CTD-lip was faster than that of CTD-lip, indicating that 11-DGA-3-O-Gal-CTD-lip might be recognized quickly by the ASGPR or GA receptor. Meanwhile, the mean plasma clearance (CL) of 11-DGA-3-O-Gal-CTD-lip (0.74 ± 0.13 L/h·kg) was higher than that of CTD-lip (0.57 ± 0.10 L/h·kg). Moreover, the mean residence time (MRT) of 11-DGA-3-O-Gal-CTD-lip (2.47 ± 1.21 h) was shorter than that of CTD-lip (5.14 ± 1.16 h). These results indicated that 11-DGA-3-O-Gal-CTD-lip was eliminated more rapidly than CTD-lip from the circulation system. In addition, the central chamber distribution volume (Vc) results for 11-DGA-3-O-Gal-CTD-lip (0.52 ± 0.15 L/kg) and CTD-lip (0.46 ± 0.19 L/kg) suggested that the modified liposomes easily distributed into tissues, which will aid in improving the therapeutic target effect. The area under the curve of drug concentration (AUC_0-∞_) of 11-DGA-3-O-Gal-CTD-lip was about 1.14 times less than that of CTD-lip (756.38 ± 15.12 μg/L·h). The AUC of 11-DGA-3-O-Gal-CTD-lip decreased as the value of CL increased, which indicated that the modification of liposomes were associated with the rapid removal of CTD from plasma. These results demonstrated that the modification of liposomes with 11-DGA-3-O-Gal had significant effects on the pharmacokinetics compared with those of CTD-lip.

### 2.8. Tissue Distribution Study

**Figure 9:**
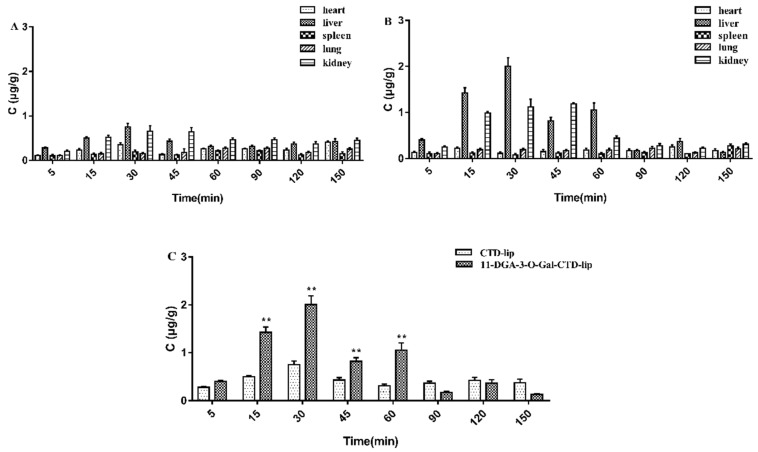
Concentration of CTD in various tissues of rats after intravenous administration (mean ± SD, n = 6). (**A**) CTD-lip; (**B**) 11-DGA-3-O-Gal-CTD-lip; (**C**) distribution of the drug content in the liver with different CTD liposomes.

The concentrations of CTD in the heart, liver, spleen, lung, and kidney were determined at various time points after intravenous administration of CTD-lip and 11-DGA-3-O-Gal-CTD-lip. The concentration-time profiles in the tissues are shown in Figure 9. These results indicate the distribution trends of the different CTD formulations in vivo for rats. We found that the liver concentration of 11-DGA-3-O-Gal-CTD-lip was significantly higher than that of CTD-lip. This result indicated that the liposomes modified with 11-DGA-3-O-Gal could deliver the drug rapidly to the liver after intravenous administration and supported our assumption that liposomes modified with 11-DGA-3-O-Gal can enhance liver-targeting through the receptor.

Furthermore, the concentration-time data were quantitively analyzed to define the liver-targeting ability. The pharmacokinetic parameters AUC_0-t_ and C_max_ in various tissues were determined, and the results are summarized in Table 4. Then, the important parameters for the evaluation of targeting ability, including targeting efficiency (T_e_), relative targeting efficiency (R_Te_), relative uptake rate (R_e_), and peak concentration ratio (C_e_), were calculated using AUC_0-t_ and C_max_. The data are listed in Table 4 and Table 5. T_e_ indicates the selectivity of a CTD formulation to target tissue. The T_e_ of CTD-lip in the liver and kidney was (26.93 ± 2.65)% and (29.98 ± 2.43)%, respectively, demonstrating that the highest selectivity rate was in the kidney.

Compared with that of CTD-lip, the T_e_ of 11-DGA-3-O-Gal-CTD-lip in liver reached (41.15 ± 3.28)%, indicating that 11-DGA-3-O-Gal was specifically recognized by receptors. Strictly speaking, the relative targeting efficiency (R_Te_) should be a comparison between the targeted preparation and the non-targeted preparation. Because cantharidin is insoluble in water, it is difficult to establish a non-targeted preparation group (cantharidin solution group), so R_Te_ is here defined as the comparison of CTD-lip and modified liposomes. The results showed that 11-DGA-3-O-Gal-CTD-lip increased the liver targeting by 1.53 times compared to CTD-lip, indicating significant liver targeting. R_e_ indicates the targeting ability of a liposomal formulation. An R_e_ greater than 1 indicates that the modified liposomes have a greater live-targeting ability than CTD-lip. The R_e_ of 11-DGA-3-O-Gal-CTD-lip was 1.69 times higher than that of CTD-lip. C_e_ indicates the effect of a liposome formulation on drug distribution. The results for the C_e_ of 11-DGA-3-O-Gal-CTD-lip were in accordance with the T_e_ and R_e_ results, demonstrating that 11-DGA-3-O-Gal-CTD-lip was more optimally recognized by the liver. Therefore, compared with CTD-lip, the addition of modified molecule improved the selectivity of the liposomes to the liver and exhibited active targeting to the liver.

## 3. Materials

Cantharidin was purchased from Xi’an Tongze Biotechnology Co., Ltd. (Xi’an, China). 11-Deoxyglycyrrhetinic acid was synthesized in our laboratory (Hunan University of Chinese Medicine, Changsha, China). Soybean lecithin was purchased from LIPOID Brand (Köln, Germany). Cholesterol was obtained from Sinopharm Chemical Reagent Co., Ltd. (Shanghai, China). Chloroform was obtained from Hunan Huihong Reagent Co., Ltd. (Changsha, China). Sephadex G-50 was obtained from GE Healthcare (Little Chalfont, Buckinghamshire, UK). 3-(4,5-Dimethylthiazol-2-yl)-2,5-diphenyltetrazolium bromide (MTT)was obtained from Biosharp (Hefei, China). The Cycletest™ Plus DNA Reagent Kit and Annexin V-FITC/PI Apoptosis Detection Kit were purchased from Becton, Dickinson and Company (Franklin Lakes, NJ, USA).

## 4. Methods

### 4.1. Synthesis of 11-DGA-3-O-Gal

**Scheme 1:**
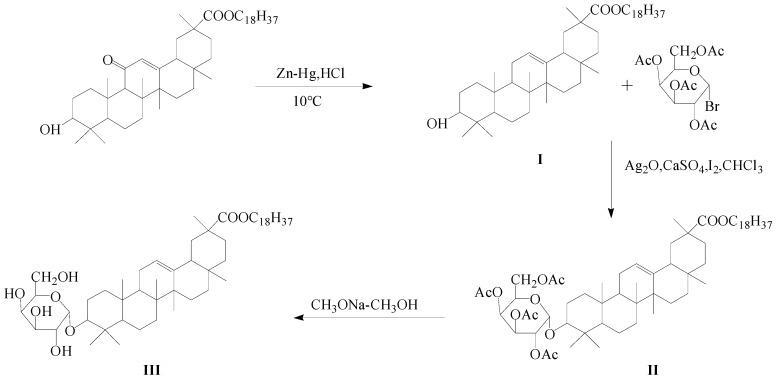
Synthesis of 11-DGA-3-O-Gal. (**I**) DGA; (**II**) 3-acetylation-galactosidase-30-stearyl deoxyglycyrrhetinic acid; (**III**) 11-DGA-3-O-Gal.

11-DGA-3-O-Gal was synthesized using DGA(I) as a hydrophobic segment and Gal as a hydrophilic segment. DGA (Scheme 1I) was synthesized by Clemmensen reaction. Zn-Hg (0.45 g) in dioxane were added to the GA (0.72 g) solution. Then 1.5 mL HCl (12 mol/L) were slowly added within 30 min, the mixture was stirred at 10 °C for 3 h. TLC was used for identification in a silica gel G plate with ethyl acetate-petroleum ether (1:5) as a developing solvent. The obtained solution was placed in a beaker, and distilled water was added to induce precipitation. The obtained precipitate was collected by filtration and dried at 40 °C. The crude products were purified by silica gel column chromatography with ethyl acetate-petroleum ether (1:5) as a eluent. The yield of DGA was 86.32% and the purity was greater than 98%.

Acetobromo-α-d-galactose (3.3 g), Ag_2_O (3.8 g), CaSO_4_ (8.7 g), and I_2_ (0.4 g) in CHCl_3_ were added to the DGA (4.0 g) solution. The mixture was stirred at 15 °C for 5 h. TLC was used for identification in a silica gel G plate with ethyl acetate-petroleum ether (1:5) as a developing solvent. The precipitate was collected by filtration and purified by silica column chromatography. The intermediate product (Scheme 1II) was dissolved in sodium methanol solution (5 mg/mL), followed by stirring for 12 h at 30 °C. The obtained solution was placed in a separation funnel, and distilled water was added to induce precipitation. The obtained precipitate was collected by filtration, washed with distilled water until the wash solution was neutral, and dried at 40 °C. The chemical structure of 11-DGA-3-O-Gal (Scheme 1III) was confirmed by ^13^C-NMR and ^1^H-NMR (in CDCl_3_, 600 MHz).

### 4.2. Preparation of Liposomes

Liposomes were prepared by the classic method of thin-film evaporation. Briefly, to prepare the modified liposomes, soybean phospholipids (SPC), cholesterol, and CTD were dissolved in 15 mL chloroform at a mass ratio of 10:1:1. The chloroform was then removed using a rotary evaporator to form a dry-lipid film at 55 °C under uniform speed. The lipid film was hydrated with phosphate-buffered saline (PBS, pH 6.4) under magnetic stirring at 55 °C. Then, the resulting suspension was sonicated with a probe sonicator for 30 min with a 2 s interval, which produced small unilamellar liposomal vesicles. Finally, the obtained liposome solution was extruded to pass through 0.22 μm pore size of microporous membranes to prepare the liposomes with an uniform size.

The targeted liposomes 11-DGA-3-O-Gal-CTD-lip were prepared by the post-insertion method. SPC, cholesterol, CTD, and 11-DGA-3-O-Gal were dissolved in 15 mL chloroform at a mass ratio of 10:1:1:1, and the rest operations were the same as above.

### 4.3. Physicochemical Characterization of Liposomes

The physicochemical characterization of liposomes was performed to determine the morphology, particle size, zeta potential (ZP), polydispersity index (PDI), and encapsulation efficiency (EE). The surface morphologies of CTD-lip and 11-DGA-3-O-Gal-CTD-lip were analyzed using a transmission electron microscope (TEM). The particle size, ZP, and PDI of liposomes were determined using a Zetasizer Nano ZS90 analyzer (Malvern Instruments, UK). The liposomes were diluted with distilled water at room temperature before measurement.

Encapsulation efficiency (EE%) refers the ratio of drug-encapsulated in liposome (W_liposome_) to the total amount of drug (W_total_) in the liposome preparation. The EE was determined using gel exclusion chromatography. To calculate the W_liposome_, about 0.5 mL of a liposome sample was added drop-wise to the top of the column and then passed through Sephadex G-50 and eluted with distilled water to separate the non-entrapped drug. The eluate containing the entrapped CTD was concentrated to 1 mL and then disrupted with a 9 mL methanol-acetonitrile mixture (at a ratio of 1:1, *v*/*v*) to calculate the W_liposome_. Similarly, to determine the W_total_ in a liposome sample, a 4 mL methanol-acetonitrile mixture (at a ratio of 1:1, *v*/*v*) was added to a 0.5 mL liposome suspension. After separation of the free drug from the liposomal formulation, the concentrations of W_liposome_ and W_total_ were measured by HPLC under the following conditions: BETASIL column: C18 (4.6 mm × 150 mm, 5 μm), mobile phase: acetonitrile-water (40:60, *v*/*v*), flow rate: 1.0 mL/min, wavelength: 230 nm, and injection volume: 10 μL.

EE (%) = (W_liposome_/W_total_) × 100% (1)

where W_liposome_ is the weight of drug being encapsulated in the liposomes, W_total_ is the weight of the total amount of charged drug.

### 4.4. In Vitro Release of CTD

The release of CTD from CTD-lip and 11-DGA-3-O-Gal-CTD-lip was determined at 37 °C using the dialysis bag method. First, a 1 mL liposome suspension was sealed in a dialysis bag. The bags were then placed in 300 mL of PBS buffer (pH 7.4) containing 0%, 0.25%, 0.5%, and 1% Tween-80 under magnetic stirring (360 r/min, 37 ± 0.5 °C). The release medium (1 mL) was sampled at pre-determined time intervals (5 min, 10 min, 15 min, 30 min, 1 h, 2 h, 3 h, 4 h, 6 h, 7 h, 24 h) and immediately replaced with the same volume of fresh medium. Then, 0.2 mL of octadecane methanol solution (7.5 μg/mL) as an internal standard was added to the samples, which were then passed through a microporous membrane filter with a 0.22 μm pore size. The concentration of CTD was measured using gas chromatography–mass spectrometry (GC–MS). The release experiments were conducted in triplicate. The accumulated release values for CTD-lip and 11-DGA-3-O-Gal-CTD-lip were calculated using the following formula:

Drug release percentage (%) = (W_t_/W_total_) × 100% (2)

where W_t_ represents CTD release at different time point, and W_total_ is the amount of drug in the liposomes.

### 4.5. In Vitro Cytotoxicity Assay

HepG2 cells and hepatocytes (L-02) were cultured in Dulbecco’s modified Eagle’s medium (DMEM) and Roswell Park Memorial Institute (RPMI) 1640 medium, respectively, supplemented with 10% fetal bovine serum (FBS), 1% penicillin, and 1% streptomycin. The cells were maintained in a humidified atmosphere containing 5% CO_2_ at 37 °C.

MTT colorimetric assays were used to evaluate the effect of liposomes on the survival rate of HepG2 and L-02 cells. Cells (7000 cells/well) were seeded in 96-well plates and incubated for 16 h at 37 °C. Then, the culture medium was replaced with 100 μL of medium containing CTD-lip or 11-DGA-3-O-Gal-CTD-lip at different CTD concentrations (0.05, 0.15, 0.25, 0.5, 1, 2, 3, 4, 5 μg/mL). After treatment for 24 h, 48 h, 72 h and 96 h, the cells were washed with PBS twice, and then, 100 μL MTT (0.5 mg/mL) was added to each well followed by incubation for another 4 h in the dark at 37 °C. The medium was then discarded, and 150 μL dimethyl sulfoxide (DMSO) was added to dissolve the formazan crystals by shaking in a horizontal oscillator for 15 min. The absorbance at 492 nm was measured with an automatic enzyme standard instrument. The inhibition rate (IR) of cellular proliferation was calculated as follows:

IR = 1 − (A_experimental group_/A_control group_) × 100% (3)

The half-maximal inhibitory concentration (IC_50_) values were calculated using SPSS.

### 4.6. Cell Migration Assays

The effects of CTD-lip and 11-DGA-3-O-Gal-CTD-lip on cell migration were assessed by the transwell chamber method. A transwell chamber was placed in a 24-well plate, and DMEM high-glucose medium containing 10% FBS was added to the lower chamber of the transwell. HepG2 cells in logarithmic growth phase were digested into a single cell suspension using trypsin. After washing twice in serum-free medium, the cells were counted and adjusted to a cell concentration of 1 × 10^4^ cells/mL, and 100 μL of the cell suspension was added to the inner chamber followed by incubation for 1~1.5 h. Then, aliquots of 100 μL of different concentrations of liposome solution were added to the inner chamber, and incubation was continued for 24 h. After the culture medium was discarded, the cells were washed three times with PBS and fixed with 4% paraformaldehyde solution for 20 min. The cell nuclei were stained with crystal violet (0.5%) for 30 min. The cells were then rinsed three times with PBS. Images of five random fields were taken under a microscope. The effect of liposomes on cell migration was evaluated by calculating the migration rate as follows:

Migration rate = Cell number in experimental group/Cell number in control group × 100%(4)

### 4.7. Cell-Cycle and Cell Apoptosis Detection

HepG2 cells were treated with different liposomes for 48 h. For all treatments, the respective drug concentrations were maintained at IC_50_ and 1/2IC_50_. After 48 h of incubation, the cell culture was aspirated into a suitable centrifuge tube, and cells were washed twice with PBS and then digested with 0.25% trypsin. After centrifugation at 1000 r/min for 5 min, the supernatant was discarded and the substratum was resuspended in PBS and adjusted to a cell concentration of 1 × 10^5^ cells/mL. Following the manufacturer’s protocol, the cell cycle and cell apoptosis was assessed using flow cytometry.

### 4.8. Pharmacokinetic Studies

Sprague Dawley(SD) rats (220 ± 20 g, male and female rats in equal numbers) were fed a standard laboratory diet with free access to water at a controlled temperature of 25 ± 2 °C and relative humidity of 60 ± 5% with a 12 h light/dark cycle. Animals were fed adaptively for 3d and kept fasting for 12 h with free access to water before experiments. The pharmacokinetic properties of CTD-lip and 11-DGA-3-O-Gal-CTD-lip were evaluated via determination of the CTD content in rat plasma. Eighteen experimental rats were randomly divided into three groups, the CTD-lip, 11-DGA-3-O-Gal-CTD-lip, and saline groups. Each group of rats was injected with a dose of 3 mg/kg via the tail vein. After injection, blood samples (0.3 mL) were collected from the retroorbital plexus at different time point (5, 15, 30, 45, 60, 90, 120, 150 min) and immediately centrifuged (4000 rpm, 20 min). Then, 100 μL of the upper plasma sample was added to 300 μL methanol followed by mixing for 3 min by vortex. After centrifugation (4000 rpm, 20 min), the supernatant was transferred to a centrifuge tube, and 50 μL octadecane (0.5 μg/mL) was added as an internal standard. The mixed solution was dried by nitrogen. The residue was reconstituted with 200 μL of methanol, vortex-mixed for 3 min, and then centrifuged at 12,000 r/min for 20 min (4 °C). Subsequently, the supernatant was examined using GC–MS. Pharmacokinetic data were analyzed with DPSv17.10.

### 4.9. Tissue Distributions

SD rats (220 ± 20 g, male and female rats in equal numbers) were fed a standard laboratory diet with free access to water at a controlled temperature of 25 ± 2 °C and relative humidity of 60 ± 5% with a 12 h light/dark cycle. Animals were fed adaptively 3 days and kept fasting for 12 h with free access to water before experiments. One hundred forty-four experimental rats were randomly divided into three groups: the CTD-lip, 11-DGA-3-O-Gal-CTD-lip, and saline groups. Each group of rats was injected with a dose of 3 mg/kg via the tail vein. After injection, rats were euthanized immediately at different time point (5, 15, 30, 45, 60, 90, 120, 150 min), and the hearts, livers, spleens, lungs, and kidneys were collected. Tissue samples were washed with normal saline, wiped with filter paper, weighed, and homogenized in equal volumes of normal saline (*w*/*v*). Then, 500 μL of homogenate was added to 1500 μL methanol followed by mixing for 3 min by vortex. After centrifugation (4000 rpm, 20 min), 3 mL of the supernatant was transferred to a centrifuge tube, and 250 μL octadecane (0.5 μg/mL) was added as an internal standard. These steps were performed according to the standard methods for plasma preparation in pharmaceutics. Finally, the supernatant was detected using GC-MS. The parameters were measured using a non-compartmental analysis with DPSv17.10. Based on the AUC and C_max_ data, the targeting parameters of each CTD liposome in a tissue were calculated, including T_e_, R_Te_, R_e_ and C_e_ [41]. The parameters were calculated as follows:

Te(%) = (AUC_target_/AUC_total_) × 100% (5)

R_Te_ = Te_modified liposome_/Te_CTD-lip_ (6)

R_e_ = (AUC_modified liposome_/AUC_CTD-lip_) × 100% (7)

C_e_ = [(C_max_) _modified liposome_/(C_max_)_CTD-lip_] × 100% (8)

### 4.10. Statistical Analysis

All data were generated in triplicate and expressed as the mean ± standard deviation (SD). Statistical comparisons between two groups were performed using Student’s *t*-test, and multiple comparisons were performed using one-way analysis of variance (ANOVA). All statistical analyses were performed using SPSS 21.0. Results were considered to statistically significant at *p* < 0.05.

## 5. Conclusions 

As previously described, cantharidin has a positive therapeutic effect on liver tumors, but its application has been limited by its water insolubility, low bioavailability, and high toxicity. In order to overcome these barriers of cantharidin in clinical application, we developed and characterized a novel, lipid-based nanocarrier for CTD, which can target hepatoma cells without the limitations of CTD. 

11-DGA-3-O-Gal was synthesized from DGA and bromine acetyl galactose glucoside. The chemical structure of 11-DGA-3-O-Gal was confirmed by NMR. In addition, 11-DGA-3-O-Gal was successfully incorporated into liposomes containing CTD. 11-DGA-3-O-Gal-CTD-lip had a particle size of less than 110 nm, with an EE lager than 90% and a sustained release for 24 h in vitro. To assess the new delivery system of 11-DGA-3-O-Gal-CTD-lip, we investigated the anti-cancer activities of CTD-lip and 11-DGA-3-O-Gal-CTD-lip in vitro and in vivo.

In vitro, compared to CTD-lip, 11-DGA-3-O-Gal-CTD-lip displayed higher cytotoxicity and migration inhibition on HepG2 cells, but did not increase the apoptotic rate of cells. The pharmacokinetic study in rats showed that 11-DGA-3-O-Gal-CTD-lip was eliminated more rapidly than CTD-lip. These results suggested that the CTD liposomes modified with 11-DGA-3-O-Gal are an efficient target carrier for the treatment of hepatocellular carcinoma. Furthermore, the tissue distribution of 11-DGA-3-O-Gal-CTD-lip was investigated, and we found that the T_e_, R_Te_, R_e_ and C_e_ of CTD in the liver were higher than in other tissues, demonstrating that 11-DGA-3-O-Gal-CTD-lip had an excellent effect of liver targeting. These results supported our hypothesis that liposomes containing 11-DGA-3-O-Gal ligand, is a potential drug delivery carrier for hepatic diseases.

## Figures and Tables

**Table 1:** Sizes, polydispersity index (PDI), zeta potential (ZP), and encapsulation efficiency (EE) of different types of liposomes (n = 3).

Type of Liposomes	Particle Size (nm)	PDI	Zeta Potential (mV)	EE %
Cantharidin (CTD)-lip	102.54 ± 3.01	0.30 ± 0.02	−3.07 ± 0.12	91.52 ± 1.61
11-DGA-3-O-Gal-CTD-lip	107.17 ± 6.12	0.26 ± 0.04	−3.59 ± 0.14	90.63 ± 1.66

**Table 2:** Fitting of each CTD liposome in in vitro release profile.

Type of Liposomes	Weibull Equation	R^2^
CTD-lip	y = 111.0038 × (1 − $e^{-\left[{\left(\frac{x-0.090986}{0.954411}\right)}^{0.230087}\right]}$)	0.9903
11-DGA-3-O-Gal-CTD-lip	y = 97.51614 × (1 − $e^{-\left[{\left(\frac{x-0.079263}{0.229073}\right)}^{0.251179}\right]}$)	0.9964

**Table 3:** Pharmacokinetic parameters in rat plasma after intravenous administration of CTD-lip and 11-DGA-3-O-Gal-CTD-lip (mean ± SD, n = 6).

Parameters	CTD-Lip	11-DGA-3-O-Gal-CTD-Lip
AUC_0-t_ (μg/L·h)	595.53 ± 17.91	509.34 ± 15.94
AUC_0-∞_ (μg/L·h)	756.38 ± 15.12	665.11 ± 19.65
T_1/2α_ (h)	0.25 ± 0.08	0.18 ± 0.09
T_1/2β_ (h)	4.86 ± 2.01	2.42 ± 1.03
K_12_ (L/h)	1.56 ± 0.87	1.90 ± 0.76
K_21_ (L/h)	0.45 ± 0.04	0.77 ± 0.23
Cl (L/h/kg)	0.57 ± 0.10	0.74 ± 0.13
Vc (L/kg)	0.46 ± 0.19	0.52 ± 0.15
MRT (h)	5.14 ± 1.16	2.47 ± 1.21

**Table 4:** Pharmacokinetic parameters of CTD-lip and 11-DGA-3-O-Gal-CTD-lip in rat tissues (mean ± SD, n = 6).

Parameters	Tissue	CTD-Lip	11-DGA-3-O-Gal-CTD-Lip
AUC_0-t_ (μg/L·h)	Heart	41.38 ± 4.27	27.18 ± 3.26
	Liver	61.88 ± 5.94	104.63 ± 3.96
	Spleen	26.93 ± 3.56	18.25 ± 5.03
	Lung	30.70 ± 4.65	26.50 ± 5.37
	Kidney	68.90 ± 3.90	77.68 ± 3.74
Cmax (μg/g)	Heart	0.35 ± 0.04	0.11 ± 0.23
	Liver	0.75 ± 0.08	2.01 ± 0.18
	Spleen	0.19 ± 0.04	0.08 ± 0.04
	Lung	0.15 ± 0.03	0.19 ± 0.03
	Kidney	0.65 ± 0.13	1.12 ± 0.17

**Table 5:** Targeting parameters of CTD-lip and 11-DGA-3-O-Gal-CTD-lip in rat tissues (mean ± SD, n = 6).

Parameters	Formulation	Heart	Liver	Spleen	Lung	Kidney
T_e_%	CTD-lip	18.01 ± 3.29	26.93 ± 2.65	11.72 ± 1.77	13.36 ± 2.87	29.98 ± 2.43
	11-DGA-3-O-Gal-CTD-lip	10.69 ± 2.04	41.15 ± 3.28	7.18 ± 2.16	10.42 ± 1.73	30.55 ± 2.03
R_Te_	11-DGA-3-O-Gal-CTD-lip	0.59 ± 0.17	1.53 ± 0.31	0.61 ± 0.12	0.78 ± 0.25	1.02 ± 0.35
R_e_	11-DGA-3-O-Gal-CTD-lip	0.66 ± 0.26	1.69 ± 0.37	0.68 ± 0.23	0.86 ± 0.25	1.13 ± 0.36
C_e_	11-DGA-3-O-Gal-CTD-lip	0.31 ± 0.08	2.68 ± 0.12	0.42 ± 0.10	1.27 ± 0.32	1.72 ± 0.23
